# Ensuring data protection for eye images by combining fine-tuned image synthesis and anonymity assessment

**DOI:** 10.1371/journal.pdig.0001487

**Published:** 2026-07-23

**Authors:** Sebastian Uschmann, Cord Spreckelsen, Sven Festag

**Affiliations:** Institute of Medical Statistics, Computer and Data Sciences, Jena University Hospital, Jena, Germany; Liverpool John Moores University - City Campus: Liverpool John Moores University, UNITED KINGDOM OF GREAT BRITAIN AND NORTHERN IRELAND

## Abstract

Advances in realistic image synthesis have led to several downstream applications in healthcare, particularly the anonymisation of biometric images. However, generative models pose significant privacy risks when synthetic outputs closely resemble the real, personal images required for training. To address these challenges, we propose a two-stage framework for generating privacy-preserving synthetic eye images based on a dataset of real photographs. Our system consists of a fine-tuned text-to-image synthesiser based on Stable Diffusion (v1-5), followed by a privacy risk scorer termed the “Cone of Privacy” (CoP). Inspired by the DreamBooth method, the synthesiser incorporates information about the original dataset to generate similar realistic yet diverse images. To mitigate identity leakage, the CoP score measures the identifiability of synthetic images with respect to the training images based on embeddings from a pre-trained vision transformer. In evaluations on a dataset of 2,000 images from 704 subjects, our approach successfully generated realistic images while filtering high-risk samples, achieving a privacy threat detection rate above 97%. The Fréchet Inception Distance (FID) between the original and the fully synthetic datasets was 57.1, with privacy-violating synthetic images scoring 51.3 and privacy-preserving images scoring 82.6, demonstrating a trade-off between realism and privacy protection. These results suggest the framework is a promising solution for anonymising biometric eye images in clinical and research applications.

## Author summary

Protecting patient privacy is essential when sharing medical images, particularly biometric data such as iris photographs. These data are critical for developing and validating diagnostic algorithms, teaching clinical skills, and enabling reproducible research. However, many tasks in research and education do not require access to original patient data, and high-quality synthetic images can provide a practical and safer alternative. Although modern AI image generators can produce highly realistic synthetic images, they may inadvertently replicate identifiable features from real individuals.

In this work, we introduce a two-part framework designed to address this challenge. First, a fine-tuned diffusion model generates realistic images of the human iris from text prompts specifying the desired features. Second, a novel privacy evaluation method, the Cone of Privacy, assesses whether a generated image can be uniquely linked to an individual in the input patient data, which could enable reidentification. Images that pose a privacy risk are automatically identified and excluded.

This approach enables researchers and educators to access high-quality, clinically meaningful eye images without revealing personal identities, providing a practical pathway for safe and privacy-preserving data sharing.

## 1. Introduction

### 1.1. Problem description and goals

The significant advances in the fields of realistic image synthesis have given rise to several downstream applications in the healthcare domain. Especially synthesis tools with the ability to produce samples approximately following the underlying real image distribution are of interest for this domain. The downstream applications span educational purposes, probabilistic anomaly detection, image segmentation and reconstruction, each contributing to improved clinical workflows, diagnostics, and training [1–5]. In many cases, the synthesised images are considered non-identifying, which would make them suitable for publication without privacy concerns [6–8]. However, several studies have shown that, contrary to this assumption, synthesised data can indeed be linked to identifiable individuals [9,10].

The present work focuses on the synthesis of biometric image data, particularly images depicting the outer structure of the eye, including identifiable iris patterns. A central concern in this context is the preservation of privacy, as biometric data of this nature are inherently sensitive and, if not properly anonymised, can lead to significant privacy risks. Consequently, the handling and processing of such data are governed by stringent privacy regulations, including the General Data Protection Regulation (GDPR) in the European Union [11] and several laws in the United States of America, such as the Biometric Information Privacy Act [12] and the Health Insurance Portability and Accountability Act [13]. These regulatory constraints often necessitate that all data processing and model training be conducted within a secure in-house environment, preventing data sharing and limiting the availability of large-scale training datasets and computational resources.

This work presents an approach to generate synthetic eye images that are both realistic and safe for public use, with minimal risk of re-identification. This system was designed to 1) be adaptable to data from different modalities, 2) learn guiding or conditioning context for the generation and 3) use a model-agnostic privacy risk scorer.

### 1.2. Related work

Two frameworks for (pseudo-)anonymous image synthesis have been extensively applied over the past few years: Generative Adversarial Networks (GANs) [14,15] and diffusion models [16–18]. GANs are trained to implicitly represent the distribution of the training data, and generate images accordingly during inference [19]. Recently, there has been a shift towards diffusion models, which generate images by gradually refining random noise into a structured image by reversing a Markov process [20].

Many synthesis methods, especially those based on diffusion, pose a significant privacy risk, as they can generate images that closely resemble individuals in the training set, which is caused by image memorisation [10,21–24]. Several author groups have suggested metrics to quantify the (level of) memorisation of such models [10,25]. The problem with image memorisation seems to occur especially when using conditioned and fine-tuned models [26,27], hence jeopardizing anonymous image synthesis based on these methods.

Truong et al. extensively summarise published works on attack scenarios targeted at diffusion models and strategies to mitigate these risks [28]. To counter membership inference attacks, which include all attacks on the privacy of individuals present in the training data, the authors identified three key strategies presented in the literature: Training regularisation, training to enforce differential privacy, e.g., differentially private stochastic gradient descent (DP-SGD), and knowledge distillation.

Regularisation, however, leads either to a significant degradation in synthesis performance or does not counter memorisation effectively [10,28]. DP-SGD is closely related to regularisation approaches and shows similar problems. It does not allow for a fine-grained utility-privacy trade-off but only for one extreme (utility without privacy or vice versa) [29].

The approaches proposed by Fernandez et al. [17] and Packhäuser et al. [18], which can be summarised as knowledge distillation, are close to the pipeline described in the present paper. However, the approaches have two fundamental issues that are tackled by the present work. The first problem is the fixed distance threshold used by both author groups. The decision whether a synthetic image is too similar to a real training image is based on a distance function and a fixed threshold. This approach neglects the fact that synthetic images that are close to many training images (embedded in dense neighbourhoods) are less likely to reveal identifiable characteristics than synthetic pictures that are close to only a single training instance. Hence, the choice of threshold value should be adaptable to these situations; however, previous approaches do not support dynamic threshold values in this sense. 2.1.2 details the privacy scorer and the dynamic thresholding approach underpinning the present methodology.

The second problem arises from the networks used to compute the distance between two images. The trained Siamese networks do not necessarily evaluate visual similarity between two input images but rather the identity of the underlying subject from which the images were taken. If only few instances are used during training, slight non-semantic variations to a training image might lead to the output of a large distance when queried together with the original training image. Consequently, synthetic images that closely resemble an original biometric image may be incorrectly classified as anonymous. To address this, we propose a distance computation based on a model pre-trained on large-scale image datasets, which compares semantic similarity rather than subject identity.

As noted by Truong et al., the privacy implications and defences against membership inference attacks in fine-tuned diffusion models are still unresolved issues [28]. In this study, we aim to address these open problems.

## 2. Materials and methods

The proposed system is presented in Section 2.1, and the corresponding evaluation study is introduced in Section 2.2.

### 2.1. System for anonymous eye image generation

**Fig 1:**
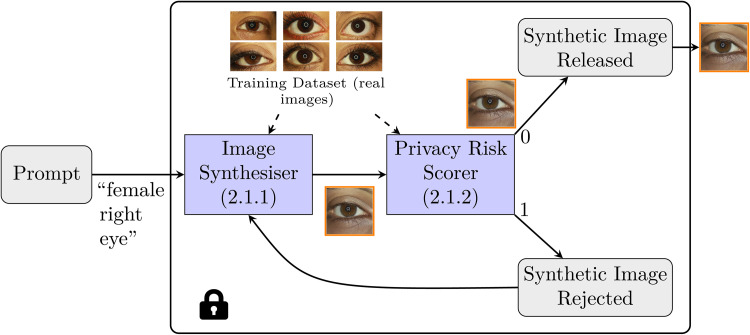
Overview of the proposed framework. The user sends a prompt defining the context of the synthesised image (and the guidance scale). Afterwards, a corresponding image is synthesised with the fine-tuned synthesiser before the result is assessed for privacy violation with respect to the training data. The illustration shows how image synthesis and privacy risk assessment interact to identify privacy-critical real images in this process. The outlined region marked with the lock symbol denotes the secure environment. The eye images are AI-generated using a fine-tuned version of Stable Diffusion 1.5, as described in Section 2.1.1.

The proposed system comprises two core building blocks, the *synthesiser* and the subsequent *privacy risk scorer* as presented in Fig 1. The first part is designed to produce high-quality synthetic images of human eyes. The scorer is needed to minimise the risk of publishing a synthesised image that can be linked to a single original training image and thus could infringe privacy. All images produced by the synthesiser are assessed by the privacy risk scorer and only if the score equals zero, corresponding to a low risk of re-identification, it is provided to the querying user. The two-part design allows for simple modification or even addition of building blocks. The only information that needs to be shared between the two blocks are the original training images. On the one hand, they are used for the fine-tuning, and on the other hand, they are needed to score the privacy risk that arises with a potential publication of the synthesised images.

The pipeline has been designed as an in-house process. Fine-tuning the image synthesiser and generating fingerprints for the privacy risk scorer necessitates the availability of the original images. A typical use case scenario would be, for example, a hospital that has patient-related images and wishes to use them to generate anonymous images of depicted pathologies. The pipeline then functions as an anonymisation service. It is imperative to note that only synthesised images with a sufficiently low risk score are released. In particular, the fine-tuned model files must remain within the secure in-house environment.

#### 2.1.1. Image synthesiser.

The synthesiser is based on a text-to-image model that is fine-tuned to produce eye images with a high level of detail. The fine-tuning procedure is inspired by the *DreamBooth* approach proposed by Ruiz et al. [30]. The parameters of the diffusion model *Imagen* that performed best in the DreamBooth experiments are not shared publicly. Hence, we decided to use the diffusion-based model that achieved the second-best performance in the experiments conducted by the DreamBooth team and for which parameter values are available, namely *Stable Diffusion* (v1-5), as the starting point of our approach [20].

The aim of the synthesiser described in our approach is slightly different to that of DreamBooth. The latter was designed to integrate information about one specific subject into the model in order to extract new images of it. For the task of anonymous image synthesis this characteristic is undesirable. The goal in the present case is to integrate information about whole object classes, like eye images captured with specific modalities, into the pre-trained model, while preserving its output diversity. It is important that the model draws images from the output distribution that are not too close to the original images but at the same time lie in an area with large probability mass, meaning they look realistic and are representative.

At the core of the DreamBooth approach lies the additive prior-preservation loss term that is added during the fine-tuning steps. In contrast to the naive fine-tuning without additional loss terms, this procedure enforces that the prior output distribution, which describes the distribution of the original diffusion model after training, is not altered drastically. Such drastic changes, which can manifest themselves in language drifts and areas of high probability density concentrations around the fine-tuning images, have been observed in the context of fine-tuning [30,31]. The complete fine-tuning loss term can be expressed as follows:


$$\begin{array}{c} {𝔼}_{\left(\underset{―}{x},\underset{―}{c})\sim P_{\text{data}}(𝐱,𝐜),\underset{―}{t}\sim 𝒰(\left\{1,\ldots ,T\right\}),\underset{―}{\epsilon }\sim 𝒩(0,𝐈\right)}\left(\underset{―}{w_{t}}\cdot \|f_{\theta }(\underset{―}{\alpha_{t}}\cdot \underset{―}{x}+\underset{―}{\sigma_{t}}\cdot \underset{―}{\epsilon },\underset{―}{c})-\underset{―}{x}{\|}_{2}^{2}\right)+\\ \lambda \cdot {𝔼}_{\underset{―}{x}\sim P_{\text{prior}}(𝐱|c_{p}),\underset{―}{t^{\prime }}\sim 𝒰(\left\{1,\ldots ,T\right\}),\underset{―}{\epsilon }\sim 𝒩(0,𝐈)}\left(\underset{―}{w_{t^{\prime }}}\cdot \|f_{\theta }(\underset{―}{\alpha_{t^{\prime }}}\cdot \underset{―}{x}+\underset{―}{\sigma_{t^{\prime }}}\cdot \underset{―}{\epsilon },\underset{―}{c_{p}})-\underset{―}{x}{\|}_{2}^{2}\right) \end{array}$$ (1)

where:

- $f_{\theta }$: conditional diffusion model
- *P*_data_: Joint distribution of images **x** and text embeddings **c**
- $\underset{―}{\epsilon }\sim 𝒩(0,𝐈)$: Standard Gaussian noise
- $\underset{―}{t^{\prime }}\sim 𝒰(\left\{1,\ldots ,T\right\})$: Uniformly sampled diffusion time step
- $P_{\text{prior}}(𝐱|c_{p})$: Pre-trained distribution conditioned on prompt “A photo of eye”
- $\underset{―}{\sigma_{t}}$, $\underset{―}{\alpha_{t}}$, $\underset{―}{w_{t}}$: Step-dependent functions

This loss is similar to the loss used in DreamBooth. The conditioning context vector **c** changes during training not because of the adaptation of the text encoder (which is kept fixed in our case) but due to the different unique tokens that are used during training. Opposed to the DreamBooth fine-tuning, not a single pair consisting of a unique token and a visual representation of specific object instance is “added” to the generative distribution of the diffusion model. Rather several pairs are added during the fine-tuning for the task of anonymous eye image generation. Each pair consists of a unique token and a visual representation of a sub-class of eye images.

This approach has two rationales. The first one corresponds to the fact that the DreamBooth method aims to generate non-anonymous identifiable images of a particular subject during inference time. By combining eye images of different subjects to one unique token during training, the probability of creating an image resembling a subject from the training set during inference is reduced. The second reason for using the outlined adapted approach is to add more fine-grained controllability of the fine-tuned model. That is, during inference, it should be possible to prompt the diffusion model to produce eyes similar to those of females or males, and either the left or right eyes from the training set. For the task at hand, the fine-tuning was not performed in a few-shot way based on 3–5 images as was suggested by the DreamBooth team [30]. It was done with a few thousand images of several hundred subjects. In comparison to the sizes of the training sets used for the Stable Diffusion models which comprise several hundred millions to several billion images [20], this is still a tiny set.

#### 2.1.2. Privacy risk scorer.

Due to the known characteristic of diffusion-based models to memorise training data and reconstruct them during inference, a second building block is added to the proposed framework: the *Cone of Privacy* (CoP) scorer, which extends the concept of the Privacy Scorer proposed by Kuppa et al. [9]. In contrast to the Kuppa score, the CoP requires only the newly generated image and the fingerprints of the real dataset, rather than the entire set of synthetic images, in order to reach a privacy decision.

It is a further mechanism to prevent privacy violations when using the framework. During the training phase of the framework it is presented all identifiable images that are used for the fine-tuning of the image synthesiser. Only with respect to these images, or rather the corresponding persons, the anonymity of synthesised images is evaluated. This is in line with the definition of “personal data rendered anonymous” in Recital 26 of the European GDPR. For all these training images semantic fingerprints are computed. To this end, the vision transformer (ViT) model [32], pre-trained on ImageNet-21k (14 million images, 21,843 classes) at a resolution of 224×224, and fine-tuned on ImageNet 2012 (1 million images, 1,000 classes) at resolution 384×384 [33], is used. The image representations computed by the trained ViT $v_{\gamma }$ serve as fingerprints of the corresponding images. This representation is denoted by **y** in the description of Dosovitskiy [32]. Since the ViT uses a fixed position embedding of image patches, it might be the case that rotated and/or scaled images are mapped to fingerprints which are not close to the embedding of the original image. Hence, not only a single fingerprint is computed for every training image, but nine. One corresponds to the original image, the others to rotated and scaled variants (rotation angles: 45°, 135°, 225°, 315°, scaling factors: 1.0, 2.0).

To analyse the influence of the image embedding method, we conducted additional experiments using fingerprints produced by the DINOv2 feature extractor [34]. For these experiments, the “giant” version (ViT-g backbone) trained on 142 million images was utilised. Unless specified otherwise, the function $v_{\gamma }$ refers to the lightweight Vision Transformer model; however, it can be replaced by other feature extractors, as demonstrated during the experiments with DINOv2.

During the inference phase, whenever a new synthetic eye image $\hat{𝐱}$ is generated, its fingerprint $v_{\gamma }(\hat{𝐱})$ is compared against all fingerprints of the training set. To this end, the cosine distance is used. As demonstrated and visualised by Black et al. this similarity computation is based on comparisons of large patterns and pose similarity between the two images [35]. For the final decision whether the publication of image $\hat{𝐱}$ poses an unacceptable privacy risk, we propose the CoP score. It is based on the cosine distance as calculated above and further combines the concepts of *eidetic memorisation* [10] and the well-established *l-diversity* privacy score [36], which focuses on maintaining plausible deniability with regard to personal identifiability. The CoP score equals 1 if the publication of the corresponding image **x** is a privacy threat to at least one individual present in the training set 𝕐 and 0 otherwise.

The key idea underlying the CoP score is to avoid enforcing a fixed minimum distance between a synthetic image and potentially sensitive training samples. Instead, the notion of similarity is defined in a relative and data-dependent manner, which can be summarised as non-uniqueness of nearest neighbour. Specifically, a synthetic image is not considered a privacy risk if its closest match in the training set is not uniquely distinguishable, but rather embedded within a sufficiently large subset of samples exhibiting comparable similarity.

This ensures that no individual training sample can be singled out as the most likely source of the generated image, thereby preserving plausible deniability. For example, a synthetic image may closely resemble a real individual without constituting a privacy threat, provided that many other individuals in the dataset exhibit a similar degree of resemblance. In this way, the approach explicitly permits generated samples that interpolate between multiple training instances, rather than requiring them to be uniformly distant from all real images.

The CoP is formally defined as follows:


$$\begin{array}{c} {CoP}_{l,r}(𝐱,𝕐):=\left\{ \begin{array}{c} 0\text{ if }\exists \delta \in \mathbb{R}[\forall 𝐲\in 𝕐\;d_{\cos}(v_{\gamma }(𝐱),v_{\gamma }(𝐲))\geq \delta \wedge \\ \;\;\exists S\subseteq 𝕐\;[|S|=l\;\wedge \\ \;\forall y^{\prime }\in S\left[d_{\cos}(v_{\gamma }(𝐱),v_{\gamma }(y^{\prime }))\leq (1+r)\cdot \delta \right]]]\\ 1\text{ else } \end{array}\right. \end{array}$$ (2)

with $l\in {\mathbb{N}}_{>0}$ and $r\in {\mathbb{R}}_{>0}$ being hyper-parameters and $d_{\cos}(𝐱,𝐲)$ the cosine distance between **x** and **y**. The larger *l* the more real images from 𝕐 have to be nearly (defined by *r*) as similar to **x** as it closest neighbour for **x** being evaluated as no privacy risk (${CoP}_{l,r}(𝐱,𝕐)=0$). To determine whether the definition evaluates to 0 for a generated image *x*, we identify the *l* nearest original images *S* and compute their distances to *x*. Let δ denote the minimum distance, corresponding to the most similar origina*l* image. We then check whether all images in *S* have distances to *x* of at most (1+r)δ.

Based on the *CoP* score, the *CoP* ratio (*CoPR*) for datasets can be defined. It allows to quantify the privacy risk posed by a whole set of synthetic images 𝕏 with respect to the training data 𝕐:


$$\begin{array}{c} {CoPR}_{l,r}(𝕏,𝕐):=\frac{1}{\left|𝕏\right|}\cdot \sum_{x\in 𝕏}{CoP}_{l,r}(𝐱,𝕐) \end{array}$$ (3)

### 2.2. Experiments

For the evaluation of the presented framework we focused on the generation of anonymous images of the outer eye structures including eyelids, sclera plus conjunctiva (white of the eye plus its covering transparent membrane), iris (circular coloured area around the pupil), cornea (clear curved layer in front of the iris), and pupil. Such views are used during many ophthalmological examinations, for example in order to check for symptoms of conjunctivitis (inflammation of the conjunctiva) [37]. Additionally, images of these structures are relatively simple to acquire without using invasive or specialised equipment. They can be taken with usual cameras and light sources or with specialised slit lamp machines. While anonymous images related to different (ophthalmological) modalities can be synthesised with the described framework, images of the outer eye structure are easiest to evaluate by non-professionals regarding realism.

#### 2.2.1. Dataset.

The image synthesiser was fine-tuned with 2000 eye images randomly sampled from the Iris Super Resolution Dataset published by Aryanmehr and Boroujeni [38]. This collection contains 4,210 images from 704 university students (392 females). For most students this dataset includes 3 images of the right eye and 3 images of the left eye (for 5 students fewer images are available).

For the fine-tuning each of the 2000 images was paired with the text embedding of “A photo of <token> eye” where <token> was one of the following four: “iML” (male left eye of the above mentioned distribution), “iMR” (male right), “iFL” (female left), “iFR” (female right). These pairs follow the distribution $P_{\text{data}}(𝐱,𝐜)$ which is used in Equation 1. All images were centre-cropped to 2048×2048 pixels and resized with bilinear interpolation to 800×800 pixels. This preprocessed image dataset is denoted by ${𝕏}_{\text{Train}}$ hereafter. For re-training of the diffusion model, the images were also normalised (mean: 0.5, std: 0.5) per colour channel.

Additionally, sample images **x** following $P_{\text{prior}}(𝐱|c_{p})$ were needed for the fine-tuning. To get this sample, we synthesised 100 images of resolution 800×800 pixels by prompting the original Stable Diffusion model with the query “A photo of eye” (20 denoising inference steps, guidance scale: 7.5).

#### 2.2.2. Ethics statement.

This study comprised two components: the development and evaluation of a data synthesis pipeline using publicly available data, and a qualitative user study. For the synthesis pipeline, we utilized the publicly available Iris Super Resolution Dataset published by Aryanmehr and Boroujeni [38]. The dataset was originally collected with appropriate ethical approval and informed consent at the respective institution. As our work involved only the secondary analysis of fully anonymised, publicly available data, no additional ethics approval was required. The user study served as a pilot study for an ongoing study using prospectively collected data covered by the approval of the ethics committee of Jena University Hospital under ID 2025–3672-BO-A. The study was conducted in accordance with the principles of the Declaration of Helsinki.

#### 2.2.3. Fine-tuning and data synthesis.

The fine-tuning was executed with the help of the modified DreamBooth training script published on Hugging Face. Specifically, the data loading process and the varying prompt input were modified to align with the description in Section 2.2.1. The fine-tuning was conducted for one epoch (with respect to 2000 real images) with an 8-bit variant of the AdamW optimizer (constant learning rate: $3\cdot 10^{-6}$, $\beta_{1}$=0.9, $\beta_{2}$=0.999, $\epsilon =10^{-8}$, decoupled weight decay: 10^−2^).

After fine-tuning the diffusion model, 50 images of resolution 800×800 pixels for every < token> and guidance scale pair were generated again with 20 denoising inference steps. The following guidance scale values were tested: 2.5, 3.0, ..., 5.0, 7.5, 10.0, 12.5, or 15.0. In total, 2000 eye images were generated for the synthetic dataset $\hat{𝕏}$.

#### 2.2.4. Anonymity scoring.

In order to evaluate the usefulness of the CoP score and the privacy risk posed by the synthesis model, the following experiments were conducted. To ensure that all identifiable individuals in the training set were represented equally for the privacy evaluation, a new set ${𝕏}_{\text{p}}$ with one picture of the left and one of the right eye (except for one student for whom only one image of the left eye is available) for each of the 704 students was created. The same preprocessing as for ${𝕏}_{\text{Train}}$ was used.

Additionally, the images in ${𝕏}_{\text{p}}$ were augmented by rotating (${𝕏}_{\text{p, rot}}$), flipping (${𝕏}_{\text{p,flip}}$) and cropping (${𝕏}_{\text{p,crop}}$). The rotations were done randomly between 10° to 350° (padded with black) and the flipping axis was also chosen randomly. The cropping was done with a centred 600×600 pixel mask (padded to 800×800 with black). Since most of these augmentations do not enhance the privacy, most of the modified raw images should lead to a CoP score of 1 when compared with ${𝕏}_{\text{p}}$ and reasonable parameter values for *l*,*r*. The following parameter values were used during the experiments: l∈{i∣20≤i≤1000,i∈ℕ},r∈{0.01·i∣0≤i≤70,i∈ℕ}.

The previously described experiments were also conducted for the case that the CoP score was computed with the DINOv2 fingerprints. These experiments help to identify the impact of the used image embedding method.

Furthermore, the CoP scores (with ViT fingerprints) for all synthetic images in $\hat{𝕏}$ with respect to ${𝕏}_{\text{p}}$ were computed. The tested parameter value pairs were chosen based on the results regarding the augmented training dataset. Since the combination *l* = 60, *r* = 0.17 led to good identification rates (>97%) of privacy threats in these sets (cf. Fig 4), it was also used to evaluate the synthetic dataset.

To assess the realism of the generated synthetic images, two additional datasets were defined, ${\hat{𝕏}}_{\text{v}}$ and ${\hat{𝕏}}_{\text{nov}}$. The set ${\hat{𝕏}}_{\text{v}}$ contains all synthetic images $\hat{𝐱}\in \hat{𝕏}$ that pose a privacy threat, i.e., $CoP_{60,0.17}(\hat{𝐱},{𝕏}_{\text{p}})=1$, while ${\hat{𝕏}}_{\text{nov}}$ contains all synthetic $\hat{𝐱}$ with $CoP_{60,0.17}(\hat{𝐱},{𝕏}_{\text{p}})=0$ ($|{\hat{𝕏}}_{\text{v}}|=1348,|{\hat{𝕏}}_{\text{nov}}|=652$). In order to retrospectively assess the degree of realism of the images generated by the pipeline, Fréchet Inception Distance (FID) [39] based on the Inception v3 model was computed between ${𝕏}_{\text{p}}$ and ${\hat{𝕏}}_{\text{v}}$, between ${𝕏}_{\text{p}}$ and ${\hat{𝕏}}_{\text{nov}}$, and between ${𝕏}_{\text{p}}$ and $\hat{𝕏}$ to analyse the realism and diversity of the synthesised images.

To complement the quantitative evaluation, we conducted a small-scale qualitative user study to assess the perceptual utility of the generated images in a downstream setting. As no standard quantitative metric exists for perception-driven tasks such as teaching, this study serves as an initial exploratory assessment. The study served as a pilot study for an ongoing study using prospectively collected data.

A total of 24 optometry students participated in a survey-based evaluation. Participants were randomly assigned to one of four groups, each corresponding to a different guidance scale used during image generation. The study consisted of two phases. In Phase 1, participants were not informed that the presented images were synthetic. Instead, a cover task was employed to avoid priming effects: participants were asked to rate the redness of the depicted eyes and to indicate whether the images were suitable for teaching. In Phase 2, participants were informed that the images were synthetic. They were then asked to classify each image as real or synthetic and to identify, for each synthetic image, the closest matching real image from a set of candidates. The presentation order of images was randomised.

## 3. Results

**Fig 2:**
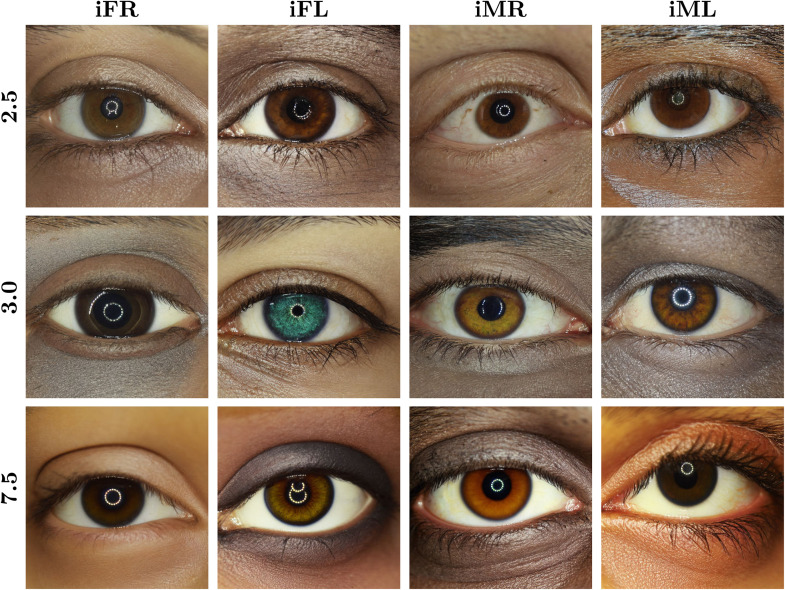
Synthesised images that do not violate the privacy of training images in ${𝕏}_{𝐩}$ (i.e., examples of images in ${\hat{𝕏}}_{nov}$). The columns represent different <token> values used during synthesis and the rows correspond to different guidance scale. The images are AI-generated using a fine-tuned version of Stable Diffusion 1.5, as described in Section 2.1.1.

**Fig 3:**
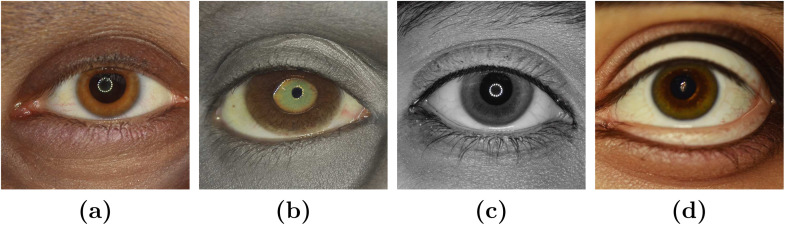
Common error cases of the synthesis. The leftmost example (a) shows one nose bridge to the left and one to the right of the eye, example (b) has two nested iris structures and similar to (c) misses colour information. Image (d) shows nested eye lid structures which are anatomically incorrect. The images are AI-generated using a fine-tuned version of Stable Diffusion 1.5, as described in Section 2.1.1.

A selection of synthetic images from ${\hat{𝕏}}_{\text{nov}}$ is shown in Fig 2. Some failure cases are represented in Fig 3.

**Fig 4:**
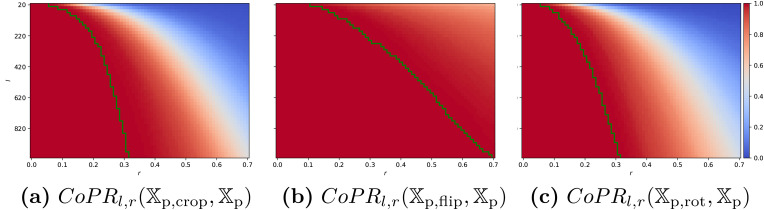
CoP ratios, computed with ViT fingerprints, of the three augmented versions compared to the non-augmented version. The rows represent the *l* values and the columns the *r* values used to compute the scores. All entries to the left of the green border line are equal to 1, indicating that all privacy risks of the calibration set are detected with the corresponding parameter combination.

**Fig 5:**
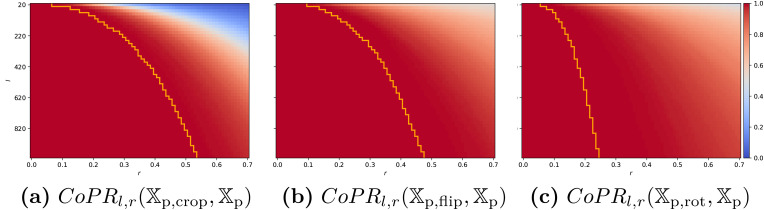
CoP ratios, computed with DINOv2 fingerprints, of the three augmented versions compared to the non-augmented version. The rows represent the *l* values and the columns the *r* values used for score computations. All entries to the left of the orange border line are equal to 1, indicating that all privacy risks of the calibration set are detected with the corresponding parameter combination.

The CoP ratios for ${𝕏}_{\text{p,rot}}$, ${𝕏}_{\text{p,flip}}$, and ${𝕏}_{\text{p,crop}}$ with respect to ${𝕏}_{\text{p}}$ are represented in Fig 4 (ViT fingerprints) and in Fig 5 (DINOv2 fingerprints). The scores regarding the synthetic images are summarised in Table 1. Under the smallest and the largest guidance scales the least privacy-violating images were generated. However, visual inspection suggests that guidance scales exceeding 10.0 produce overly colourful and artistic images lacking biological plausibility.

**Table 1:** *CoPR*_60, 0.17_ scales for the subsets of $\hat{𝕏}$ defined by the <token> used in the prompt (rows) and the guidance scale (columns) with respect to the real images in ${𝕏}_{𝐩}$.

Prompt	Guidance scale									
token	2.5	3.0	3.5	4.0	4.5	5.0	7.5	10.0	12.5	15.0
iFL	0.66	0.74	0.84	0.76	0.84	0.94	0.88	0.80	0.70	0.40
iFR	0.48	0.74	0.72	0.66	0.70	0.78	0.74	0.66	0.52	0.24
iML	0.56	0.66	0.70	0.72	0.72	0.80	0.76	0.66	0.56	0.62
iMR	0.46	0.62	0.64	0.72	0.72	0.76	0.64	0.72	0.64	0.48

The side and sex condition encoded into the text prompt is learned by the model as depicted in Fig 2. Nonetheless, a proportion of synthetic images appear to depict the incorrect side, or alternatively, exhibit a combination of both sides, as the inspection of the nose bridge(s) shows (compare Fig 3(a)). However, it should be noted that assessing the correctness is not always straightforward. Nevertheless, we conducted a manual sanity check on 100 generated images (guidance scale 3.0), assessing whether the specified side was correctly generated, and observed inconsistencies in 13 of the samples.

The FID between ${𝕏}_{p}$ and the full synthetic set $\hat{𝕏}$ is 57.1. Between ${𝕏}_{p}$ and the set of privacy-violating images ${\hat{𝕏}}_{\text{v}}$ the FI distance is 51.3, while the distance from the set of real images to the non-violating images in ${𝕏}_{\text{nov}}$ is 82.6.

To complement the quantitative evaluation, the results of the qualitative user study are summarised as follows. Images generated with a guidance scale of 2.5 were most frequently rated as suitable for teaching, with approximately 85% of participants in this group providing positive assessments. In the identification task, participants selected the closest real image with an accuracy of approximately 50%, corresponding to chance-level performance. Furthermore, participants showed limited ability to reliably distinguish between real and synthetic images, with classification performance remaining close to chance level.

## 4. Discussion

### 4.1. Critical appraisal

The experiments demonstrate that the proposed combination of a fine-tuned image generation model and the CoP score constitutes a practical anonymisation framework for biometric image data. It was shown that several clinically relevant conditioning factors (e.g., side and sex) can be integrated during fine-tuning and that the CoP score is a useful metric to automatically assess re-identification risk in (pseudo-)anonymised images.

The image embedding method used for the CoP computations has a strong influence on the final score. However, neither method demonstrated overall superiority. While the ViT fingerprints achieved better privacy risk detection on flipped images, the DINOv2 fingerprints yielded higher detection rates on the cropped dataset. Regardless of the embedding function used, calibrating the CoP scorer for each dataset to be protected, as illustrated in Figs 4 and 5, is crucial for practical and secure applicability. This context dependence aligns with recent jurisprudence of the European Court of Justice (Case C-413/23 P), which emphasises that the classification of data as personal or anonymous depends on the means reasonably available for re-identification and may vary with the perspective of the data controller [40]. In this sense, identifiability is not an absolute property but a relative, context-dependent notion, which closely aligns with the underlying principle of the CoP framework, where re-identification risk can be controlled and calibrated to application-specific requirements. However, the proposed framework does not provide formal privacy guarantees in the sense of differential privacy, but instead follows a data-dependent, risk-based approach.

The applicability of two embedding methods shows that the CoP framework is not inherently tied to a specific embedding or distance metric. Instead, it can be flexibly adapted by substituting alternative feature representations or similarity measures, which may be beneficial for domain-specific privacy risk assessment. Similarly, the proposed pipeline is not tied to a specific generative model and can be extended by integrating alternative image generation approaches, enabling future comparative evaluations within the same framework.

To ensure the proper functioning of the privacy scorer, it is essential that identifiable individuals are equally represented in the reference dataset used for the CoP score. By adjusting the parameters of this score, the trade-off between re-identification risk, realism, and fidelity can be controlled, as reflected by the FID values. It is important to note that FID should not be interpreted in isolation in the context of privacy-preserving image synthesis. While lower FID values typically indicate higher visual fidelity, they may also correspond to increased similarity to real images and thus elevated re-identification risk. Moreover, strong optimisation towards low FID can reduce sample diversity. In contrast, the proposed framework explicitly balances fidelity, diversity, and privacy risk. In this context, very low FID values may even indicate insufficient anonymisation.

The qualitative user study further informs the assessment by providing insights into the perceptual realism of the generated images. These findings suggest that lower guidance scales (in particular 2.5) yield synthetic images that are perceived as sufficiently realistic for educational purposes, and participants showed limited ability to reliably distinguish synthetic from real images. At the same time, the near chance-level performance in identifying the closest real image indicates that commonly used embedding distances (e.g., cosine distance in ViT feature space) may not accurately reflect human perceptual similarity.

Although the model learns conditioning factors such as side and sex, they are not yet consistently enforced during generation. Some generated samples exhibit inconsistencies, particularly with respect to side. However, all generated samples remain within the target domain of eye images, and the deviations primarily affect finer-grained attributes. Future work will focus on improving conditioning reliability and extending the framework to incorporate additional clinically relevant attributes, such as disease-specific characteristics.

However, the computational demands of the proposed approach due to considerable discard rates of synthetic images and the dependency on semantic-aware embedding systems must be taken into account. The rejection of synthetic images with high privacy risk results in wasted computing power. To reduce this, we will consider incorporating privacy-aware fine-tuning methods as a complementary addition to our approach.

### 4.2. Limitations

A limitation of the present study is the acquisition of monocentric, monoethnic data and the limited number of conditioning context values. In future studies, it is planned to gather data from diverse modalities as well as supplementary meta-information concerning the patients/volunteers. This will allow a more comprehensive assessment of the proposed framework’s capability to handle more intricate data and context combinations. Moreover, it is envisaged that clinical experts will evaluate the synthesised results regarding their usability for educational and research purposes.

A direct quantitative comparison with alternative approaches was not feasible with our dataset. Potentially suitable baselines (particularly the approaches by Fernandez et al. [17] and Packhäuser et al. [18]) require training data that cannot be constructed due to a lack of feature variability inherent to our application domain (iris images). Other baselines were not directly comparable because they operate on substantially different image resolutions, or did not achieve sufficient fidelity in our setting, or could not be implemented due to the lack of available open-source code.

An additional limitation is the small sample size and exploratory design of the qualitative user study. While the results provide initial insights into the perceptual realism and practical utility of the generated images, they are not sufficient to draw statistically robust conclusions. Larger-scale studies with domain experts and task-specific evaluation protocols are required for a more comprehensive assessment.

Furthermore, the conditioning on clinically relevant attributes (e.g., side and sex) is not perfectly preserved in all cases, which may limit the reliability of the generated images for certain downstream applications. In safety-critical or educational settings, this suggests that an additional layer of expert review may be beneficial to ensure the correctness of clinically relevant features.

### 4.3. Conclusion

The approach establishes a pipeline to be used within a protected environment from which any number of synthetic images can be generated on demand. Through fine-tuning, the model can be adapted to different image domains and conditioning factors.

The pipeline successfully addresses the problems raised at the outset: 1) The Cone of Privacy (CoP) avoids fixed thresholds when selecting privacy-critical images. The dynamic thresholds take into account the fact that the risk of re-identification decreases the more similar original images of different people are found in the vicinity of the synthetic image. 2) The approach does not require that several sufficiently different training images be available per person, which is particularly advantageous for biometrically stable features such as iris images.

In summary, the results indicate that the presented approach effectively balances privacy protection and data utility, making it a promising solution for anonymising biometric image data in clinical and research settings.
